# Polyphenols and Human Beings: From Epidemiology to Molecular Targets

**DOI:** 10.3390/molecules26144218

**Published:** 2021-07-12

**Authors:** Celestino Santos-Buelga

**Affiliations:** Grupo de Investigación en Polifenoles (GIP-USAL), Universidad de Salamanca, E-37007 Salamanca, Spain; csb@usal.es; Tel.: +34-923-294-500

Dietary polyphenols have been associated with health benefits in the prevention of a range of degenerative and age-related diseases that constitute the major causes of death and incapacitation in developed countries. The first observations of the beneficial effects of food polyphenols were made by the Nobel laureate Albert Szent-Györgyi and coworkers in the 1930s, describing the ability of flavonoids extracted from lemon juice and paprika to counteract vascular failure associated with ascorbic acid deficiency [1,2]. Those findings led them to consider flavonoids as a vitamin (i.e., vitamin P) [3,4], a consideration that was maintained for several years until it was demonstrated that they were not indispensable [5]. Later on, in the 1990s, the interest in polyphenols was renewed with observations derived from the Zutphen and Seven Countries epidemiological studies, showing the existence of inverse relationships between the dietary intake of flavonoids and the risk of coronary heart disease [6,7]. Afterwards, a great deal of observational studies in different countries and population groups followed, leading to similar conclusions on the positive relation between consumption of flavonoids or polyphenols and protection against cardiovascular diseases in addition to other chronic conditions such as type II diabetes, different cancers, or neurodegenerative diseases such as Alzheimer’s and Parkinson’s diseases (see [8] for a review). Nowadays, it is considered that phenolic compounds contribute, at least in part, to the protective effects of plant-rich diets, and the elucidation of their role in human nutrition has become a relevant issue in food and pharmacological research. 

Evidence contributed by the epidemiological studies is, however, insufficient to make undisputed claims about the positive health effects of polyphenol consumption. Most of the available information on the biological activity and effects of these compounds has been obtained from model and animal studies, and there are still many gaps in the knowledge of their actual effects on human health and the subjacent involved mechanisms. Further research must still be conducted on studying aspects such as bioavailability, pharmacokinetics, biological targets, mechanisms of action, actual bioactive compounds, active doses, and possible adverse effects. Moreover, long-term, randomized, controlled dietary intervention trials are required to assess the unequivocal role that polyphenols may play in preventing human disease [9].

For years, the beneficial health effects of polyphenols have been associated with their antioxidant capacity, largely demonstrated in vitro. Thus, they are acknowledged as efficient scavengers of most types of oxidizing species, via mechanisms that involve the transfer of an H atom or a single electron to a stabilizing radical [10]. Furthermore, the ability of many polyphenols to form stable complexes with metal ions, such as iron or copper, that can act as catalyzers in the production of oxidant species (e.g., OH^•^, O_2_^•^ˉ H_2_O_2_) is also recognized [11].

Nonetheless, when considering the in vivo situation, it is necessary to consider that the effects of polyphenols not only depend on their intrinsic antioxidant and scavenging activities, but are also strongly influenced by their bioavailability. Following consumption, polyphenols can be subject to modifications in the upper section of the gastrointestinal tract; be absorbed and biotransformed in the small gut; or, more often, reach the gut, where they will interact with colon microbiota and be catabolized into a range of phenolic metabolites that might be absorbed and distributed to different biological targets via systemic circulation. The high variety of polyphenol structures, their bioavailabilities, and the different molecular mechanisms of action involved, together with the interindividual variability in composition and activity of gut microbiota, are all variables that influence the in vivo effects of polyphenols [12]. Ultimately, only small amounts of polyphenols are absorbed, with the majority being biotransformed in the organism, so that their ability to reach tissue, cell, and molecular targets is very limited, and their levels in such media are very low. Thus, the final levels of phenolic metabolites that can be found in human plasma are usually situated in the nanomolar or low micromolar range [13], concentrations that are far below those of other dietary and physiological antioxidants such as urate, α-tocopherol, ascorbate, and glutathione [14].

What seems clear is that the notion of polyphenols acting as ‘systemic’ antioxidants is unlikely to be the main explanation for their putative health effects. Other hypotheses have emerged, and novel biological targets have been identified that might contribute and explain the in vivo activity of polyphenols. Nowadays, polyphenols are being increasingly recognized to exhibit a pleiotropic character, affecting multiple molecular targets and intracellular signaling cascades, most of them interconnected [15]. For instance, they have been proposed to act as modulators of gene expression and signaling pathways related to cell function and protection. Among others, some polyphenols and metabolites were shown to be able to activate the antioxidant response element (ARE) and regulate the oxidative status of the cell by modulating the activity of oxidative enzymes such as xanthine oxidase, protein kinase C, and nitric oxide synthases [8].

The eleven high-quality research and review articles comprising this Special Issue look into the biological activities and effects of polyphenols in the context of different health and disease conditions. Diverse methodological approaches are used to delve into the molecular mechanisms subjacent to polyphenol effects, including assays in specialized cell lines [16,17], microorganisms [18], model organisms such as the nematode *C. elegans* [19], or mice models [20], as well as molecular docking studies upon potential target proteins [20], involving distinct polyphenols, either as pure compounds [19] or diverse and complex phenolic mixtures [16,18,20,21]. In addition, interactions with non-phenolic bioactives, such as cannabinoids [17,22], humulones and lupulones [22], or polyunsaturated fatty acids [23] are explored.

The published papers describe a range of activities for polyphenols, including antioxidant [19,23], anti-inflammatory [20,24], anticancer [21], neuropreventive [16], analgesic and antipyretic [20], estrogenic [22], and antiviral [18]. Further, the therapeutic potential of this family of compounds for the prevention or treatment of distinct human diseases, beyond their recognized cardiovascular protective effect, is shown, such as for bowel disorders [25], breast cancer [21], prostatic hyperplasia [24], and ocular degenerative diseases [26]. All in all, these articles provide evidence-based support for the versatility and potential of polyphenols to be used as bioactive compounds for nutritional, nutraceutical, and pharmacological purposes for the prevention or mitigation of a diverse range of human metabolic conditions.

Nevertheless, despite the amount of accumulated knowledge, the mechanisms involved in the in vivo activity of polyphenols are not yet fully understood and may vary from one compound to another. Much work is still required to define their precise biological targets, as well as to further deepen the understanding of aspects such as bioavailability, the role of gut microbiota on polyphenol metabolism and resulting effects, or interactions among different phenolic compounds and/or with matrix components to ascertain the impact of polyphenols in the organism. Significant advances in all these issues should be expected in the near future, taking into account the number of studies that are currently in progress around the world together with the more powerful analytical techniques and novel methodological approaches at our disposal, including omics tools.
